# Self-Perceived Individual Health Responsibility Among Healthcare Workers: Associated Factors and Relationship with Preventive Behaviors—A Cross-Sectional Study

**DOI:** 10.3390/healthcare14152330

**Published:** 2026-08-01

**Authors:** Ocxana Maria Țocan, Larisa Pinte, Alexandru Marian Constantin, Cristian Băicuș, Anna Schneider-Kamp, Marina Ruxandra Oțelea

**Affiliations:** 1 Doctoral School, “Carol Davila” University of Medicine and Pharmacy, 050474 Bucharest, Romania; ocxana-maria.tocan@drd.umfcd.ro; 2 Clinical Department 5, “Carol Davila” University of Medicine and Pharmacy, 050474 Bucharest, Romania; larisa.pinte@umfcd.ro (L.P.); cristian.baicus@umfcd.ro (C.B.); marina.otelea@umfcd.ro (M.R.O.); 3 Clinic of Internal Medicine, Colentina Clinical Hospital, 020125 Bucharest, Romania; 4 Department of Business & Management, University of Southern Denmark, 5230 Odense, Denmark; anna@sam.sdu.dk; 5 Clinic of Occupational Medicine, Colentina Clinical Hospital, 020125 Bucharest, Romania

**Keywords:** individual health responsibility, health literacy, preventive behaviors, health promotion, lifestyle risk factors, screening participation in healthcare personnel

## Abstract

**Background/Objectives:** Empowerment strategies promoting healthy behaviors are important for preventing chronic diseases and cancer, while individual health responsibility (IHR) plays a significant role. Although widely discussed in the literature, the concept of IHR does not have clearly defined domains or a validated instrument for assessment. Its overlap with locus of control theory, self-motivation, and personality type creates further difficulties. Meanwhile, perception of one’s own responsibility for health is an intuitive, simple item to answer, reflecting a person’s belief related to this topic. Moreover, for ethical reasons, this concept cannot be separated from personal beliefs and should not be adjusted according to strict scales and externally imposed definitions. Therefore, this study aimed to: (1) explore whether there is an association between self-perception of IHR and preventive/risk-taking behaviors; and (2) examine socio-demographic factors associated with the IHR score and three self-perceived characteristics, namely self-perceived locus of control, health literacy, and health status. **Methods:** A cross-sectional study with 968 healthcare workers was conducted in a large, multidisciplinary hospital covering several behaviors (smoking, alcohol consumption, diet, and participation in a screening program for hepatitis C). IHR was evaluated on a scale from 1 to 10, where 1 meant “to very little extent” and 10 meant “to a great extent”. **Results:** The average IHR score was 8.80 (SD = 1.88). In the univariate analysis, the IHR score was significantly related to gender, age, education, marital status, and all three personal characteristics. In the multivariate analysis, the socio-demographic associations lost statistical significance. IHR maintained its association with the healthy diet score in the multivariable model (B = 0.113, *p* = 0.036). In the unadjusted analyses, the distribution of IHR differed between participants who declared alcohol consumption and those who declared abstinence (χ^2^ = 17.157, *p* = 0.002). However, this association was not retained in the multivariable model. Self-perceived internal locus of control was negatively associated with screening acceptance, but not with IHR. The OR of 0.927 refers to a one-point increase on the 1–10 scale. Across the full observed scale range, this corresponds to an OR of 0.51 (95% CI: 0.29–0.89), indicating lower odds of screening acceptance among participants with higher perceived internal control. Because this estimate assumes linearity across the entire scale, it should be interpreted cautiously. **Conclusions:** The gap between perceived responsibility and actual behavior suggests that IHR messaging alone is unlikely to be sufficient. Future research should consider social desirability and external locus of control when analyzing IHR and health behaviors.

## 1. Introduction

In a world of apparently ubiquitous access to health information, we are still facing a high burden of preventable diseases that are among the leading causes of death worldwide, such as cardiovascular diseases, cancer, diabetes, and COPD (chronic obstructive pulmonary disease) [1]. Modifiable risk factors such as body mass index, smoking, diet, exercise, alcohol consumption, and sleep account for a high percentage of the prevalence and mortality of the above-mentioned medical conditions [2,3,4,5]. Despite this commonly accepted knowledge, primary prevention efforts to control these risk factors are considered insufficient in many European countries [6].

While guidelines emphasize the importance of doctor–patient communication in boosting patients’ motivation to adopt a healthy lifestyle [7], the results of real-life interventions are far from consistent. Personalized interventions are generally more effective, but a single interaction session has shown limited results in people with lower levels of education [8]. Longer duration (12-week) complex health literacy programs, including guidance on obtaining reliable health information, education regarding health-related behaviors, drug adherence, and sessions of motivational interviewing, have proven effective across all education-based stratification groups [9].

From a comprehensive perspective on what contributes to the maintenance or improvement of health, health responsibility involves not only the individual, but also the social network, society as a system [10], and their interactions [11]. Social and system-level determinants seem to have convergent results across various cultures, while individual health responsibility (IHR) is highly dependent on contextual specificities. On the one hand, for example, social support had a positive impact on the motivation to improve lifestyle changes or participate in screening in both individualistic and collectivist societies [12,13]. On the other hand, in a systematic review of media communication, the IHR for preventing obesity, diabetes, and cancer was mostly emphasized in Western, individualistic societies [14].

IHR is accepted mainly as a desirable prerequisite for health promotion defined as “prudential responsibility” for one’s own well-being [15] and described as “a daily, gradual, and personalized process” [16]. IHR does not exclude social responsibility, which should ensure the ethical provision of healthcare [17]. Consistent with a prudential interpretation, IHR can be understood as the capacity to make free choices within a framework of self-awareness and awareness of the consequences those choices may have for oneself and others [18].

Although distinct, IHR and the theory of locus of control have some overlap. IHR covers, besides causality (i.e., the perceived relation between behavior and disease), stability (i.e., the possibility of change) and the control of the causing factor (i.e., the extent to which the causing factor is controlled by the person or depends on external circumstances) [19]. It is therefore dependent not only on individual characteristics, but also on socio-cultural characteristics [15]. The locus of control theory includes the concept of self-efficacy proposed by Bandura and defined as “the conviction that one can successfully execute the behavior required to produce the outcomes” [20]. Based on this theory, people can be characterized by high internal control (feeling responsible for their actions) or high external control (relying more on social interaction or on “powerful others”). According to the same theory, health-related behaviors are also influenced by another element, “chance” [21].

IHR is also perceived differently by different stakeholders. In a qualitative study conducted among Swedish general practitioners, IHR assessed from the doctors’ perspective was defined as “owning your own health”, “not leaving the whole responsibility to the GP (General practitioner)”, “taking active measures to keep and improve health”, and “accepting help in health” [22]. When the patients’ perspective was investigated, it was mainly related to the interaction with the health system [23] and not to the health impact of their personal behaviors. Health promotion strategies targeting health-related behaviors may serve as a bridge between the health system’s responsibility and IHR, with proven benefits for patients with chronic diseases [24]. Therefore, it is increasingly important to understand how the self-perceived IHR relates to actual behavior in the current evolution of Internet-based healthcare [25], with an increasing number of patients searching for self-care information outside clinical encounters.

As part of a workplace preventive intervention, we analyzed the relationship between the self-perceived IHR and several actual health-related behaviors: smoking, alcohol consumption, diet, and acceptance of a screening test. Our defined aims were:

- The primary aim was to explore whether there is an association between self-perceived IHR and preventive/risk-taking behaviors.
- The secondary aim was to examine socio-demographic factors associated with the IHR score and some personal characteristics, such as self-perceived locus of control, self-perceived health literacy, and self-perceived health status. We assessed this relationship in a healthcare setting; therefore, we investigated whether there were any differences between different healthcare occupations.

**Figure 1:**
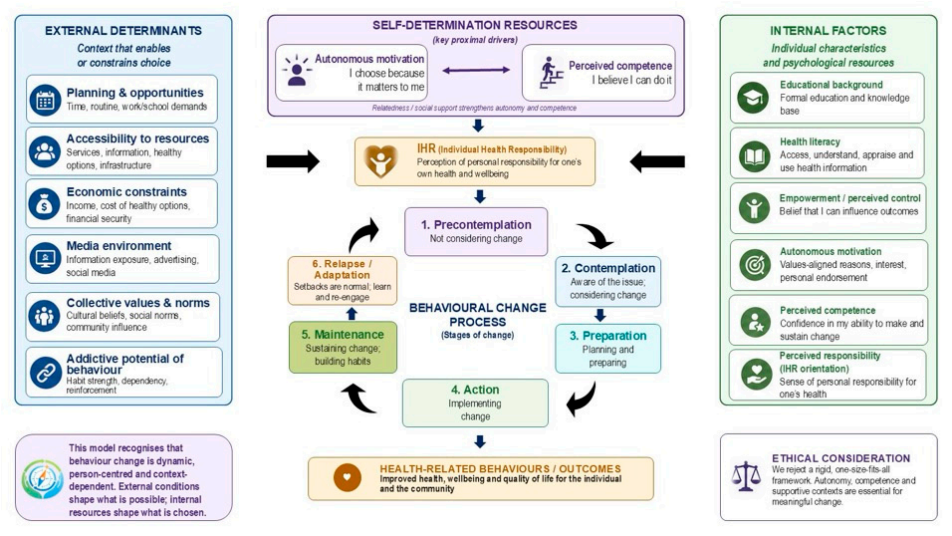
Conceptual framework.

The conceptual framework (Figure 1) of this article is based on the broader field of health behavior change. Within this framework, we have synthesized the main interactions between IHR and other personal characteristics. However, we consider that imposing a rigid frame on all the participants and all investigated behaviors would be both inappropriate and ethically problematic. Moreover, according to self-determination theory and interventions targeting behavioral change, autonomous motivation and self-perceived competence to make changes are better proxies for behavioral improvement among adults than many other intervention-related characteristics [26].

The definition of health literacy includes the ability to understand health information and make decisions based on it [27]. From this perspective, health literacy is closely related to IHR.

IHR has rarely been investigated among healthcare workers (HCWs), although they are significant promoters of healthy lifestyles and could serve as role models for others. Thus, assessing factors that influence health behaviors in HCWs might provide insights that generalize from this segment to the general population.

## 2. Materials and Methods

The analysis was conducted in a large Bucharest hospital, as part of a preventive program for cancer through the identification and treatment of persons infected with hepatitis C virus. The study included all employees willing to participate, including doctors, nurses, assistant nurses, other health professionals, and administrative and technical staff. No selection criteria were used. The study was approved by the Ethical Committee of Colentina Clinical Hospital (No. 06/26 February 2024). A full description of the program has already been reported elsewhere [28]. Compared to previous communications [29], this analysis includes data from the completed enrollment. We compared the total sample with earlier partial samples, performing sensitivity analysis to investigate whether there were relevant differences.

For the sample size estimation, we included national statistics data for alcohol consumption and smoking in the Cochran formula, using a 95% confidence level (z = 1.96) and *p* < 0.05. The result was a minimum of 245 men and 382 women for alcohol intake, and 236 for smoking. As there are no national data for diet and screening test acceptance, using an estimate of 50% conformity to the recommendations and/or acceptance, the minimum number obtained was 384. Based on these calculations, our sample size meets the statistical requirements for the significance of the results.

The IHR of the participants was evaluated exclusively based on self-perceived responsibility, using the question: “To what extent do you feel responsible for your health?”. The respondents could choose a score on a scale from 1 to 10, where 1 meant “to a very little extent” and 10 meant “to a great extent”. This was a pragmatic choice intended to test the usefulness of this approach for a large study. As explained in the introduction, self-perception of IHR is the most ethically appropriate way to evaluate a possible relationship with behaviors. For regression analysis, the recorded score was used. For descriptive comparisons, the original 1–10 IHR score was collapsed into five ordered categories.

The participants were also asked to rank their health status (scored from 1 to 4, from very healthy to very ill), their health literacy (scored 1 if comprehensive, 2 if fair, and 3 if insufficient), and their belief regarding an internal locus of control (“things happening to me”). Answers to the latter item ranged from: things happening to me are “very much independent” (scored 1) to “very much dependent on my own decisions, efforts and behaviors” (scored 10).

Several socio-demographic factors (age, gender, education, occupation, marital status, and residence) were considered as covariates.

The behavioral aspects investigated were smoking, alcohol consumption, and diet. For the smoking status, the participants were divided into current, ex-, and never smokers. Participants were considered ex-smokers if they had stopped smoking at least one year before. Alcohol consumption was assessed both as a dichotomous variable and as the number of drinks per week.

Diet was assessed for 10 major food categories (red meat, chicken, processed meat, fish, fresh fruits, fresh vegetables, cooked vegetables, dairy products, sweets, and soft drinks) and classified according to compliance with current recommendations [30,31] in terms of consumption frequency (never/less than once a month/1–3 times per month/most days of the week/every day). Optimal consumption was defined as follows: red meat 1–2 times/week, poultry on most, but not all, days/week, processed meat 1–2 times/week, fish 1–2 times/week, fresh fruits daily, uncooked vegetables daily, cooked vegetables daily, cookies less than 1/month, soft drinks less than 1/month. The score was calculated as follows: for ideal compliance, the responses were given 2 points; for acceptable compliance, 1 point; and for all other frequencies of consumption, 0 points. For example, fish intake 1–2 times/week was scored as 2; if consumed several times per week, the score was 1. Any other frequency scored 0. In both scenarios, all components were equally weighted. A total diet score was calculated as the sum of the individual scores. The number of respondents meeting the recommended consumption frequency for each food category is presented in Figure S1 (Supplementary Materials).

We also evaluated the acceptance of the screening test for hepatitis C, the preventive activity included in this program.

### Statistical Analysis

SPSS software (IBM^®^ SPSS^®^ Statistics v26.0) was used for the statistical analysis.

Descriptive analysis. Descriptive statistics were used to calculate means and standard deviations (SDs) for numerical variables, and frequencies and percentages for categorical variables. The distribution of the IHR score was also examined descriptively. For descriptive comparisons, the original 1–10 IHR score was collapsed into five ordered categories.

Univariate analysis. Variable comparisons were performed with chi-square tests for categorical variables and ANOVA, Kruskal–Wallis, or Mann–Whitney tests for numerical variables, according to their distribution. In the first step, we analyzed which of the socio-demographic factors were related to the IHR score. Subsequently, we integrated into the analysis the personal characteristics self-perceived by each respondent: the self-perceived health status, the self-perceived control over things happening, and the self-perceived health literacy. In the next step of the analysis, we related the IHR score and all other variables to the actual behaviors.

Multivariable analysis. Regression analysis, linear or binary according to the type of outcome, was performed to assess the associations. Health-related behaviors were considered outcomes: smoking, defined as non-smokers versus ever smokers and past smokers versus current smokers; alcohol consumption, defined as abstainers versus consumers and number of drinks per week; diet score; and acceptance versus non-acceptance of the screening test. For the full regression model, age, gender, education, marital status, occupation, and self-perceived scores for health status, health literacy, internal locus of control, and IHR were considered as predictors. The following variables were considered the reference categories: men for gender, rural residence, single for marital status, non-smokers for smoking, and abstinent for alcohol. Missing data were not included in the analysis.

The IHR score was treated as a continuous variable in the primary regression analyses because it was measured on a 1–10 ordered scale, and this approach preserved the full range of responses. The regression coefficients therefore reflect the association with a one-point increase in self-perceived IHR. However, because the distribution of IHR was skewed, with many participants reporting high scores, this was considered a possible ceiling effect when interpreting the results.

Sensitivity analysis. To assess whether the findings depended on treating IHR as a continuous variable, we performed a sensitivity analysis comparing participants with the highest IHR scores, defined as 9 or 10, with all other participants. Multicollinearity was assessed in all regression models: for linear regression, a VIF value > 10 was considered suggestive of multicollinearity, while for binary regression, a correlation coefficient >0.7 was considered an indicator of multicollinearity.

## 3. Results

In this study, 1194 healthcare workers were invited, and 968 (81.07%) agreed to complete the questionnaire. The main differences between participants and non-participants were younger age (participants were younger; U = 83,501, *p* < 0.001) and gender: 23.6% of men refused to participate versus 17.8% of women (χ^2^ = 4.258, *p* = 0.039).

The questionnaire was completed for 100% of responses for the majority of items, with less than 1% missing data. Data were missing only for marital status (5 missing answers) and alcohol consumption (8 missing answers). These minor missing data were unlikely to influence the results and did not require further sensitivity analysis.

**Figure 2:**
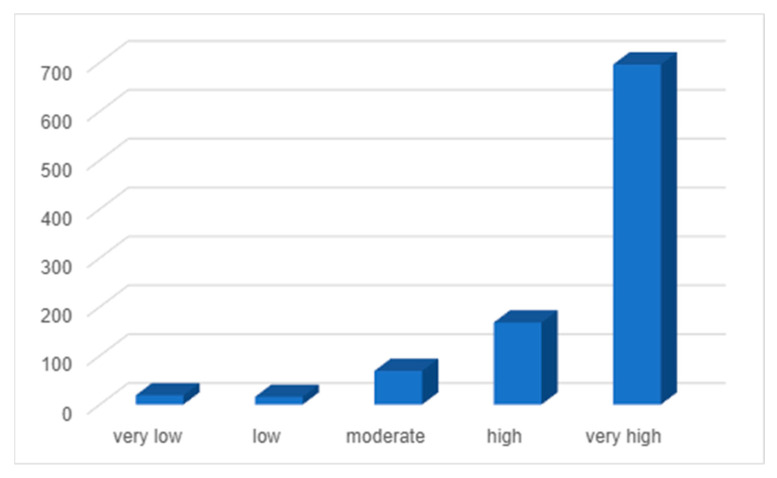
The distribution of the individual health responsibility score in the total sample.

The overall IHR score was high (mean = 8.80, SD = 1.88), with a skewed distribution, as 696 respondents (71.9% of the total sample) ranked it at a very high level (Figure 2).

The detailed score distribution according to different characteristics is presented in Supplementary Table S1. The differences in score distribution varied significantly by age, marital status, education, residence, and occupation (Table 1).

All three self-perceived characteristics showed different distributions according to the IHR score (Table 1). Because the IHR score was not normally distributed, we performed a sensitivity analysis, comparing the highest scores (9 and 10) with all others, to verify this association. In the binary regression model, the association with self-perceived health status (B = −0.494, *p* < 0.001) and self-perceived locus of control (B = 0.069, *p* = 0.009) remained highly significant, while the relation with self-perceived health literacy was marginally attenuated (B = −0.266, *p* = 0.054). There was a high correlation between self-perceived health status and the IHR, with a tendency to assume more responsibility when minor health issues are present. This association was statistically significant in both gender groups. A similar strong relation was identified between IHR and the self-perceived internal locus of control. The highest average IHR scores were observed in the extreme groups, namely in those considered to have high or very high internal locus of control. The sensitivity analysis by gender showed significant differences, with no statistically significant association (*p* = 0.124) in the men’s group and a statistically significant relation in the women’s group (*p* = 0.017).

When the socio-demographic factors and the personal characteristics were used as covariates, all socio-demographic factors were no longer significantly associated with the IHR score (Table 2). Overall, these variables explained 5.6% of the IHR variance (R^2^ = 0.056, *p* < 0.001).

Some of the health-related behaviors, such as alcohol consumption (including the number of drinks per week) and smoking, were directly associated (Supplementary Table S2) (*p* = 0.01), while smoking and the total diet score were inversely related (*p* = 0.01). There was also an indirect correlation between the total diet score and the number of drinks/week (*p* = 0.05). Smoking was also related to a lower adherence to the recommended diet, as the average diet total score in non-smokers (3.55) was significantly higher than in ever smokers (3.23) (*p* = 0.007).

The distribution of the IHR score for the health-related behaviors is presented in Supplementary Table S3. There were 472 ever smokers in this cohort, of whom 81 had stopped smoking more than one year ago. In the simple regression models, IHR scores did not differentiate between ever smokers and non-smokers (B = −0.24, *p* = 0.523) nor between current and ex-smokers (B = 0.009, *p* = 0.361). In the multivariate analysis, ever smoking was inversely associated with education and directly associated with urban living; men were also significantly more likely to report this habit (Table 3). No multicollinearity was detected for the variables included in the analysis. A Hosmer–Lemeshow test confirmed that the model (χ^2^ = 12.261, *p* = 0.140) was adequate.

In a sensitivity analysis (doctors versus all other participants), ever smoking was less frequent in doctors (41%) compared to all other job categories (51.9%), (χ^2^ = 8.972, *p* = 0.003). In the multivariate analysis (full model), being a doctor was associated with a higher chance of never smoking (B = 0.615, *p* = 0.018).

Alcohol-related analyses were based on self-reported data. Accordingly, the binary outcome was defined as declared alcohol consumption versus declared abstinence, and the continuous outcome was defined as the reported number of drinks per week. In unadjusted analysis, the IHR score was distributed differently between those declaring alcohol consumption and abstainers (χ^2^ = 17.157, *p* = 0.002). In the multivariate regression, alcohol consumption was related to gender, age, and smoking (Table 4). No multicollinearity was noticed, and the Hosmer–Lemeshow test confirmed the model (χ^2^ = 11.312, *p* = 0.185).

The reported number of drinks per week was low overall and differed across IHR categories, with the highest mean value observed in the relatively high IHR category, as shown in Supplementary Tables S4 and S5.

**Figure 3:**
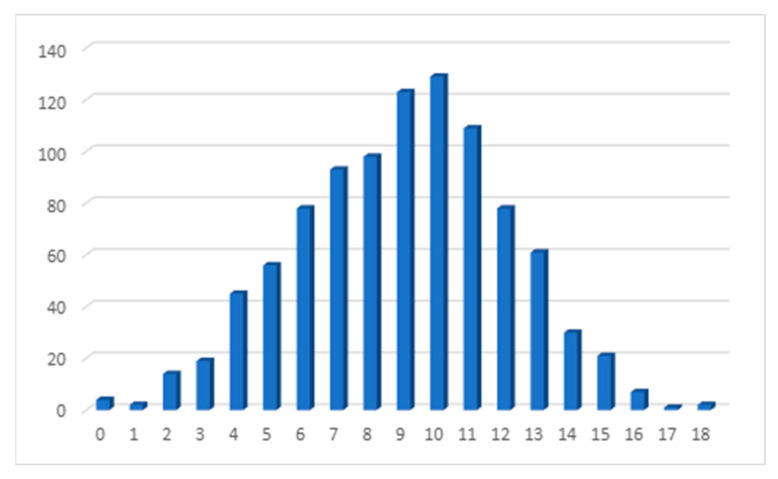
The distribution of the diet score in the total sample.

The average diet total score was 8.95 (SD = 3.103) from a maximum of 18. Only 2 participants reached the maximum score. The score distribution was approximately normal (Figure 3), and the residual analysis confirmed this.

Better scores in the univariate regression were identified for women (B = 0.604, *p* = 0.018) and older age (B = 0.052, *p* < 0.001). In the univariate regression, the total diet score was marginally associated with IHR (B = 0.097, *p* = 0.069). In the multivariate analysis, the association between the total diet score and IHR became statistically significant (Table 5). Other covariates significantly associated with diet score were age, education, marital status, and perceived internal locus of control. No multicollinearity was detected between these variables.

The perception of having a healthy diet was directly related to the diet score (B = 0.588, *p* < 0.001).

From the total sample, 771 participants (79.6%) accepted the screening test. IHR was not associated with screening acceptance, whereas self-perceived internal locus of control was negatively associated with participation in the full regression model (Table 6).

## 4. Discussion

The main finding of our study is that the high self-perceived IHR in a sample of healthcare workers is consistently related to age, self-perceived health status, self-perceived health literacy, and self-perceived internal locus of control, but shows limited association with health-related behaviors. The high IHR score in this population probably reflects the awareness of prudential and proportional responsibility, which is expected to be present in a healthcare organization [32,33].

The link between IHR and age is consistent with the more general finding that conscientiousness, one of the Big Five psychological traits incorporating responsibility, is related to age [34]. Applying the investigation of conscientiousness to the health domain, a large national study in the United States confirmed that responsibility in adults is directly related to age [35]. In another study focused on well-being and risk of accidents, a significant relationship between age and the reduction in unhealthy behaviors was also found [36].

The relation with the perceived health status was more nuanced and showed an interesting pattern: for people with no disease or with a serious disease, the responsibility seemed to depend on external factors, while for the ones with minor health issues, for whom lifestyle interventions are most effective, the IHR was the highest. These findings suggest the high dependence of people with chronic diseases on the health system and on their social support [37,38]. Healthy behaviors are insufficiently represented in the Romanian population [29], even among those who have a direct experience with a chronic disease through a relative with a chronic illness [39], and attributing causality to external factors rather than to lifestyle-related ones is not unusual. In a review of qualitative studies, diet and exercise were not linked by healthy persons to the risk of diabetes; instead, external factors such as stress or air pollution, or non-modifiable factors, such as genetics, were emphasized [40]. This might explain the lower scores of IHR in this category of respondents.

There might also be an explanation for the higher scores of IHR in subjects with minor health issues, and this could be their better health literacy. In particular, this could reflect the knowledge about the importance placed by current guidelines on lifestyle changes. Our respondents had a high chance of having direct access (through their professional education) or indirect access (through their easy access to doctors and nurses) to these recommendations and of being aware of the personal effort necessary for compliance. As this is just an assumption, the relation between IHR and access to scientific, evidence-based information provided by healthcare professionals should be investigated further in patients with minor health issues.

IHR was strongly associated with perceived internal locus of control, an expected finding given that personal responsibility presupposes some degree of perceived control over health. This association differed by gender and was accompanied by a higher prevalence of healthy behaviors among women. A similar pattern has been reported among healthcare workers [41], whereas a study of medical students found a greater internal locus of control in men [42]. Apart from healthy diet, however, the associations between internal locus of control and the investigated health-related behaviors were absent or inconsistent. Given the large proportion of respondents with high IHR scores, the prevalence of healthy habits might also be expected to be high. This was not the case for smoking, alcohol consumption, or diet. This finding suggests that healthcare workers may be subject to the same normative beliefs and social pressures as others, despite feeling more responsible for their health. However, the high IHR scores themselves may also partly reflect professional norms and socially desirable self-presentation. We did not explore the reasons for this discrepancy, but it represents an interesting objective for future investigation.

Before discussing the alcohol consumption association, some general considerations should be considered. Alcohol intake is usually underreported in Romania in different national polls, in contrast with the country’s 5th place in the global statistics concerning annual consumption per capita [43] and its status as one of the leading causes, together with diet and air pollution, of the high rates of preventable diseases in the country [44]. In our sample, the percentage of people declaring alcohol intake was lower than in the national statistics, where it reaches 80% in men and 54.5% in women [45]. The underreporting of drinks was explained in other Romanian studies through the “perceived normative opinion of their belonging group for fear of social exclusion” [43], and alcohol stigma was found to be directly related to the actual level of consumption in several European countries [46,47].

Other studies concerning alcohol consumption have shown socio-demographic findings similar to ours; namely, that men, particularly younger men [48], and smokers are more likely to report this behavior [49,50]. In a systematic review, results from different studies showed that the perception of personal responsibility for alcohol use disorder is higher than for other diseases [51]. The tendency to assume personal responsibility was also present in our cohort.

The observed association between IHR and declared alcohol consumption should be interpreted cautiously. Given the likelihood of underreporting and social-desirability bias, especially in healthcare professionals, these findings may reflect reported drinking behavior rather than actual alcohol intake. Therefore, the results should not be interpreted as direct evidence that IHR is strongly related to true alcohol-consumption patterns. The participants in this study reported numbers of drinks per week that did not exceed, on average, the socially acceptable limits. Nevertheless, better communication of risks related to alcohol, including cancer, is necessary [52].

Smoking was not related to IHR in this sample. Smoking was more prevalent than in the general population, where it is estimated at 18.9% [53], than that reported in other healthcare studies [54]. It was probably better reported than other health-related behaviors, as attitudes toward smoking are generally neutral [55], and smoking is even associated with self-efficacy and self-esteem among young adults [56].

In a study including six European countries, including Romania, the most important predictors of quitting were health concerns, the price of cigarettes, and setting a good example for children [57]. We did not find an association with marital status, but a good understanding of the health concerns could be an explanation for the significantly lower prevalence of smoking in doctors. There were also no differences in IHR between non-smokers and smokers and between smokers and ex-smokers, which might correspond to findings that the efficacy of smoking cessation has a certain genetic influence [58,59].

Contrary to expectations, smoking was not associated with IHR. Similar findings have been previously reported, including in a meta-analysis showing no consistent relationship between internal locus of control and smoking [60]. This is also in line with the finding that self-perceived internal locus of control largely mediates the relation between locus of control and smoking [61] and could explain why self-perceived control over what happens to them was not associated with this behavior.

We did not find a strong association between diet score and IHR, although it was statistically significant in the multivariate analysis. A healthy diet is an increasing concern in the Romanian population, mainly among the higher educated, younger women, but in our sample, the average score was relatively low, with few persons in the upper end of the distribution. However, declaring interest in a healthy diet does not necessarily translate into actual consumption [62]. A significant finding in our study is the strong correlation between the actual behavior and the way respondents described their diet, supporting that the participants possessed knowledge about dietary recommendations.

It was also interesting that among all health-related behaviors, this was the only one associated not only with IHR, but also with the perceived internal locus of control. This is different from other studies, including one on healthcare personnel, where no relation between locus of control and diet was found [41,63]. Besides possible cultural habits, this difference might be explained by the different age composition of the study population. For example, in the study referring to emergency healthcare workers [41], adherence was high regardless of internal control scores, whereas in our sample, adherence was relatively low, consistent with other Romanian studies [31]. The second study investigated children’s diet and concluded that “powerful others” (namely parents, family and the internet) and “chance” are more important than internal control [63]. In the meta-analysis cited above [60], diet was related to an internal locus of control. The association of gender, age, marital status, and education with a healthy diet has been reported in many other studies [64,65,66].

Acceptance of the test was higher than in other population-based studies [67,68] and similar to that reported in another study performed in an occupational setting [69]. IHR was not related to the acceptance of the preventive test, but the self-perceived internal locus of control was associated with participation. This finding suggests that, at least for a direct medical intervention, the gap between endorsing personal responsibility for health and preventive behaviors may be related to perceived ability to control one’s own circumstances.

The correlation was negative in this sample of healthcare workers, possibly because participation was motivated by the inability to fully control occupational exposure to blood-borne infections. Unlike the other health-related behaviors examined, screening was an easily accessible preventive measure that required no behavioral change, which may explain why only perceived internal locus of control was associated with participation. This interpretation is consistent with studies linking self-efficacy and perceived autonomy to screening uptake [70]. Screening participation may also be influenced by social norms [13,71] and the fear of positive results [72].

Although the association between internal locus of control and screening acceptance was statistically significant, its interpretation remains speculative. The effect size suggests a potentially meaningful association across the locus-of-control scale, but the cross-sectional design precludes conclusions about causality or directionality. Therefore, this finding should be interpreted as exploratory and requires confirmation in future studies.

Overall, the self-perceived IHR was more strongly related to sociocultural and psychological determinants such as health literacy and internal locus of control than to actual health-related behaviors. As an exception to this, for alcohol consumption and adherence to nutritional recommendations, IHR was not a strong correlate of the investigated behaviors. This discrepancy suggests that endorsing responsibility for health does not necessarily translate into healthier lifestyles and that behavioral interventions should address contextual and motivational barriers in addition to promoting personal responsibility.

## 5. Conclusions

The prevalence of smoking, alcohol consumption, and poor diet is high even among healthcare workers. The main contribution of this study is the identification of a discrepancy between high self-perceived responsibility for health and less favorable preventive behaviors in a population expected to have higher health literacy and greater awareness of prevention. Overall, self-perceived IHR showed weak or inconsistent associations with most of the preventive health behaviors examined and appears insufficient on its own to explain these behaviors.

Alcohol consumption and poor nutrition seem to be integrated in the healthcare workers’ perception as factors related to the internal locus of control, but the relatively high prevalence of non-compliance with current recommendations supports the need for multifactorial health-promotion approaches that integrate individual, social, organizational, and environmental determinants of health behavior, even in a segment of the population with better health literacy than the general population.

The IHR-focused messaging may be worth exploring in relation to healthier diet choices, although this association should be interpreted cautiously, and such messaging should be integrated into broader multifactorial health-promotion strategies. Reinforcing the IHR message in relation to declared alcohol consumption remains uncertain but would be worth testing prospectively; based on our findings, these studies should consider social desirability, external loci of control, and other sociocultural and economic factors.

No statistically significant association was found between self-perceived IHR and smoking behavior in this sample, which is consistent with evidence that smoking cessation is shaped primarily by factors other than declared responsibility.

The association between perceived internal locus of control and screening uptake suggests that interventions addressing self-perceived autonomy and self-efficacy may be worth exploring in relation to screening participation.

## 6. Limitations

This study has several limitations that should be considered. The use of a single-item measure to assess IHR represents an important methodological limitation of this study, as it may not capture the full complexity of individual health responsibility and does not allow for an assessment of internal consistency. While a validated questionnaire might have been preferable, we consider it difficult to validate an instrument operationalizing IHR independent of the purpose (e.g., preventive behavior or compliance with medical recommendations), as IHR represents an intimate but, at least for adults, self-explanatory construct and could be perceived by the respondents as closely related to accountability for their health. Nevertheless, when using this self-perception item, the significant relationship between the IHR score and the other self-perceived characteristics (self-perceived internal locus of control and self-perceived health literacy) implies a relatively high consistency of our results.

There are various scales validated to assess responsibility in a broader sense [73,74,75,76], but none of these scales includes a domain specifically dedicated to health. Regarding the locus of control, the number of validated instruments is larger. Some use six items for the internal control domain, of which one is very similar to the question we assessed (“I am directly responsible for my health” in one form or “If I take the right actions, I can stay healthy”, in the other form). Another scale uses only three items for the internal locus of control [48]. These scales do not predict health-related behaviors [21]. Therefore, it was worth exploring whether a self-perceived single-item assessment of one’s own responsibility for health is reflected in the considered health-related behaviors.

To assess the use of a single item, we compared our results to those obtained by applying these validated scales and examining their associations with the preventive behaviors. Our results were either similar or could be explained by different sample characteristics such as age differences. The questionnaire was filled in without the investigators’ interference, and for each item assessing smoking, alcohol consumption, diet, and screening participation, there was also the option to choose the answer “I refuse to answer”. Even so, we could not exclude, and we found indications of social-desirability bias, particularly for alcohol consumption and nutrient intake. This bias might be present in any survey of self-reported data, but it is commonly documented in research concerning alcohol consumption [77] or nutritional intake [78].

Although the sample was quite large, it is a single-site sample, which limits the generalizability of the results, even in the context of other healthcare worker cohorts. Because of its cross-sectional design, this study cannot infer causality.

The fact that our study was conducted among healthcare workers has advantages and disadvantages. The main advantage is that the survey covers the self-perception of one of the important stakeholders in public health and might reflect the expectations of this segment for the general population, as reported in the Dutch survey of general practitioners [22]. This could also be seen as a disadvantage. Social desirability bias may have affected not only the self-reported behaviors, but also the main exposure variable itself. Healthcare workers may feel professionally expected to report a high level of responsibility for their own health, which could partly explain the very high IHR scores observed in this sample. Nevertheless, a counterargument is that the IHR score was not significantly different between occupations, which included persons without formal medical education. Even if the sample composition limits generalizability, our results contribute to the understanding of how to develop health promotion strategies in the Romanian population for whom alcohol consumption and poor diet are important public health concerns, as they are for most other European and many non-European populations.

## Figures and Tables

**Table 1:** Differences in the distribution of the IHR score according to sample characteristics.

Characteristic	Number (N = 968)	% of Responses Registered	Distribution of the IHR Score	
			Pearson Chi^2^	*p*
Age group			44.286	<0.001
20–30 years	195	20.1%		
31–40 years	185	19.1%		
41–50 years	344	35.5%		
51–60 years	226	23.3%		
>60 years	18	1.9%		
Gender			3.150	0.533
Women	787	81.3%		
Men	181	18.7%		
Residence			9.609	0.048
Rural	147	15.2%		
Urban	821	84.8%		
Marital status			8.112	0.088
Single	278	28.9%		
In a relation	685	71.1%		
Education			54.024	<0.001
Tertiary education	481	49.7%		
Secondary education	416	43%		
Lower secondary education	71	7.3%		
Occupation			33.266	0.007
Doctor	292	30.2%		
Nurse	394	40.7%		
Assistant nurse	182	18.8%		
Other health professionals	25	2.5%		
Administrative & technical staff	75	7.7%		
BMI			18.294	0.107
Underweight	22	2.3%		
Normal weight	419	43.3%		
Overweight	322	33.3%		
Obese	205	21.2%		
Self-perceived health status			42.053	<0.001
Perfectly healthy	413	42.7%		
Minor health issues	488	50.4%		
Serious health issues	65	6.7%		
Almost permanently ill	2	0.2%		
Self-perceived health literacy			35.900	<0.001
Comprehensive	552	57%		
Fair	389	40.2%		
Unsatisfactory	27	2.8%		
Self-perceived internal locus of control			147.951	<0.001
Very low	89	9.2%		
Low	52	5.4%		
Medium	197	20.4%		
Relatively high	252	26%		
High	378	39%		

**Table 2:** Factors associated with individual health responsibility.

	Unstandardized B	Standard Error	Standardized Beta Coefficient	t	*p*-Value	95% CI
Gender	0.047	0.154	0.010	0.306	0.760	−0.255, 0.349
Age	−0.010	0.007	−0.058	−1.530	0.126	−0.023, 0.003
Education	0.131	0.109	0.044	1.205	0.229	−0.082, 0.345
Residence	0.211	0.169	0.041	1.251	0.211	−0.120, 0.542
Occupation	0.094	0.061	0.056	1.534	0.128	−0.027, 0.214
Marital status	0.046	0.132	0.011	0.350	0.727	−0.212, 0.304
Self-perceived health status	−0.378	0.105	−0.124	−3.609	<0.001	−0.584, −0.173
Self-perceived health literacy	−0.280	0.114	−0.083	−2.465	0.014	−0.503, −0.057
Self-perceived internal locus of control	0.069	0.022	0.101	3.154	0.002	0.026, 0.111

**Table 3:** Factors associated with smoking (ever smokers versus never smokers).

	B	Standard Error	Wald	OR	*p*-Value	95% CI
Gender (women vs. men)	−0.418	0.174	5.772	0.658	0.016	0.468, 0.926
Age	−0.017	0.008	5.110	0.983	0.024	0.969, 0.998
Education	−0.586	0.125	21.856	0.556	<0.001	0.435, 0.711
Residence	0.620	0.194	10.268	1.859	0.001	1.272, 2.717
Occupation	0.089	0.069	1.650	1.093	0.199	0.954, 1.252
Marital status (married as reference)	−0.076	0.148	0.269	0.926	0.604	0.694, 1.237
Self-perceived health status	−0.50	0.119	0.176	0.952	0.675	0.754, 1.200
Self-perceived health literacy score	−0.065	0.128	0.259	0.937	0.611	0.729, 1.204
Self-perceived internal locus of control	−0.005	0.025	0.037	0.995	0.846	0.948, 1.044
Self-perceived IHR	−0.032	0.036	0.798	0.968	0.372	0.901, 1.040

**Table 4:** Factors associated with alcohol intake (consumers versus abstainers).

	B	Standard Error	Wald	OR	*p*-Value	95% CI
Gender (women vs. men)	−1.656	0.184	80.694	0.191	<0.001	0.133, 0.274
Age	−0.028	0.009	9.570	0.972	0.002	0.955, 0.990
Education	0.154	0.153	1.007	1.167	0.316	0.863, 1.576
Residence	0.427	0.252	2.871	1.533	0.090	0.935, 2.512
Occupation	−0.052	0.086	0.371	0.949	0.542	0.802, 1.123
Marital status	0.445	0.187	5.675	1.560	0.017	1.082, 2.250
Self-perceived health status	0.065	0.144	0.203	1.067	0.653	0.804, 1.416
Self-perceived health literacy	−0.003	0.157	0.000	0.997	0.985	0.732, 1.135
Self-perceived internal locus of control	0.032	0.031	1.074	1.033	0.300	0.972, 1.098
Self-perceived IHR	−0.064	0.044	2.173	0.938	0.140	0.861, 1.021

**Table 5:** Factors associated with the diet score.

	B	Standard Error	Standardized Beta Coefficient	t	*p*-Value	95% CI
Gender	0.391	0.256	0.049	1.529	0.127	−0.111, 0.893
Age	0.057	0.011	0.194	5.133	<0.001	0.035, 0.079
Education	0.531	0.181	0.107	2.935	0.003	0.176, 0.886
Residence	−0.238	0.281	−0.028	−0.849	0.396	−0.789, 0.313
Occupation	0.082	0.102	0.029	0.804	0.422	−0.119, 0.283
Marital status	0.544	0.219	0.079	2.485	0.013	0.114, 0.973
Self-perceived health status	0.125	0.175	0.025	0.714	0.476	−0.219, 0.470
Self-perceived health literacy	−0.229	0.190	−0.041	−1.206	0.228	−0.601, 0.143
Self-perceived internal locus of control	0.073	0.036	0.064	2.011	0.045	0.002, 0.145
Self-perceived IHR	0.113	0.054	0.068	2.096	0.036	0.007, 0.218

**Table 6:** Factors associated with participation in the screening test (acceptors versus non-acceptors).

	B	Standard Error	Wald	*p*-Value	OR	95% CI
Gender	0.126	0.209	0.364	0.546	1.134	0.753, 1.707
Age	−0.024	0.009	6.595	0.01	0.977	0.959, 0.994
Education	0.033	0.149	0.05	0.824	1.034	0.772, 1.385
Residence	−0.231	0.242	0.913	0.339	0.794	0.494, 1.275
Occupation	0.032	0.085	0.14	0.709	1.032	0.874, 1.219
Marital status	−0.239	0.188	1.627	0.202	0.787	0.545, 1.137
Self-perceived health status	0.283	0.149	3.602	0.058	1.327	0.991, 1.776
Self-perceived health literacy	−0.044	0.157	0.079	0.779	0.957	0.704, 1.301
Self-perceived internal locus of control	−0.075	0.032	5.671	0.017	0.927 *	0.871, 0.987
Self-perceived IHR	0.057	0.043	1.8	0.18	1.059	0.974, 1.151

* OR per 1-point increase in self-perceived internal locus of control.

## Data Availability

The data presented in this study are available on request from the corresponding author. The data are not publicly available due to privacy and ethical restrictions related to participant-level survey data.

## Supplementary Materials

The following supporting information can be downloaded at: https://www.mdpi.com/article/10.3390/healthcare14152330/s1.

## Abbreviations

The following abbreviations are used in this manuscript:

IHR	Individual health responsibility
SD	Standard deviation
COPD	Chronic obstructive pulmonary disease
GP	General practitioner
BMI	Body mass index
