# Supplementary material for: Self-Perceived Individual Health Responsibility Among Healthcare Workers: Associated Factors and Relationship with Preventive Behaviors—A Cross-Sectional Study
Source: Healthcare (Basel). 2026 Aug 1;14(15):2330. doi: 10.3390/healthcare14152330 (PMC13465295; doi:10.3390/healthcare14152330)
Supplement: Supplementary file 1 [file healthcare-14-02330-s001.zip › healthcare-4350791-supplementary.pdf]

Supplementary Table S1. Distribution of the IHR score according to the socio-demographic characteristics.

	All	Score 1	Score 2	Score 3	Score 4	Score 5
Age group***						
20-30	195 (20.1%)	0 (0%)	2 (12.5%)	8 (11.6%)	42 (25%)	143 (20.5%)
31-40	185 (19.1%)	0 (0%)	2 (12.5%)	5 (7.2%)	31 (18.5%)	147 (19.1%)
41-50	344 (35.5%)	11 (57.9%)	8 (50%)	31 (44.9%)	44 (26.2%)	250 (35.9%)
51-60	226 (23.3%)	8 (42.1%)	4 (25%)	25 (36.2%)	45 (26.8%)	144 (20.7%)
>60	18 (1.9%)	0 (0%)	0 (0%)	0 (0%)	6 (3.6%)	12 (1.7%)
Gender						0.533
Women	4.54 787 (81.3%)	17 (89.5%)	15 (93.8%)	58 (84.1%)	134 (79.8%)	563 (80.9%)
Men	4.63 (181, 18.7%)	2 (10.5%)	1 (6.3%)	11 15.9%)	34 (20.2%)	133 (19.1%)
Residence						0.048
Rural	4.46 (147 =15.2%)	3 (15.8%)	4 (25%)	18 (26.1%)	19 (11.3%)	103 (14.8%)
Urban	4.57 (821, 84.8%)	16 (84.2%)	12 (75%)	51 (73.9%)	149 (88.7%)	593 (84.8%)
Marital status						0.088
Single	278 (28.9%)	3 (16.7%)	4 (25%)	16 (23.2%)	62 (36.9%)	193 (28.9%)
In a relation	685 (71.1%)	15	12	53	106	499
Education***						
Tertiary	481 (49.7%)	5 (26.3%)	3 (18.8%)	23 3(3.3%)	102 (60.7%)	348 (50%)
Secondary	416 (43%)	12 (63.2%)	12 (75%)	37 (53.6%)	56 (33.3%)	299 30.9%)
Lower secondary	71 (7.3%)	2 (10.5%)	1 (6.3%)	9 (13%)	10 (6%)	49 (7%)
Occupation**						
Doctor	4.66 (292, 30.2%)	1 (5.3%)	2 (12.5%)	9 (13%)	70 (41.7%)	210 (30.2%)
Nurse	4.5 (394, 40.7%)	11 (57.9%)	8 (50%)	36 (52.2%)	58 (34.5%)	281 (40.4%)
Assistant nurse	4.49 (182, 18.8%)	5 (26.3%)	5 (31.3%)	17 (24.6%)	24 (14.3%)	131 (18.8%)
Other health professionals	4.52 (25,	1 (5.3%)	0 (0.0%)	1 (4%)	6 (24%)	17 (68%)
Administrative & technical staff	4.61 (75,7.7%)	1 (5.3%)	1 (6.3%)	6 (8.7%)	10 (6%)	57 (8.2%)
BMI						0.107
Underweight	22 (2.3%)	0 (0%)	0 (0%)	1 (1.4%)	2 (1.2%)	19 (2.7%)
Normal weight	419 (43.3%)	8 (42.1%)	5 (31.3%)	20 (29%)	82 (48.8%)	304 (43.7%)
Overweight	322 (33.3%)	8 (42.1%)	9 (56.3%)	33 (47.8%)	45 (26.8%)	227 (32.6%)
Obese	205 (21.2%)	3 (15.8%)	2 (12.5%)	15 (21.7%)	39 (23.2%)	146 (21%)
Self-perceived health status **						
Perfectly healthy	413 (42.7%)	2 (10.5%)	2 (12.5%)	22 (31.9%)	58 (34.5%)	329 (47.3%)
Minor health issues	488 (50.4%)	15 (78.9%)	12 (75%)	38 (55.1%)	89 (53%)	334 (48%)
Serious health issues	65 (6.7%)	2 (10.5%)	2 (12.5%)	9 (13%)	21 (12.5%)	31 (4.5%)

Permanently ill	2 (0.2%)	0 (0%)	0 (0%)	0 (0%)	0 (0%)	2 (0.3%)
Self-perceived health literacy***						
Comprehensive	552 (57%)	6 (31.6%)	6 (37.5%)	27 (39.1%)	93 (55.4%)	420 (60.3%)
Fair	389 (40.2%)	13 (68.4%)	7 (43.8%)	38 (55.1%)	70 (41.7%)	261 (37.5%)
Unsatisfactory	27 (2.8%)	0 (0%)	3 (18.8%)	4 (5.8%)	5 (3%)	15 (2.2%)
Control over things happening***						
Very low	89 (9.2%)	2 (10.5%)	0 (0)%)	7 (10.1%)	5 (3%)	75 (10.8%)
Low	52 (5.4%)	2 (10.5%)	7 (43.8%)	9 (13%)	8 (4.8%)	26 3.7%)
Medium	197 (20.4%)	3 (15.8%)	6 (37.5%)	26 (37.7%)	44 (26.2%)	118 (17%)
Relatively high	252 (26%)	2 (0.8%)	2 (0.8%)	8 (3.2%)	76 (30.2%)	164 (65.1%)
High	378 (39%)	10 (52.6%)	1 (6.3%)	19 (27.5%)	35 (20.8%)	313 (45%)

Supplementary Table S2. Correlations between health-related behaviors.

		alcohol	Non-smokers vs. ever smokers	Total score diet	No. of drinks/week
**Alcohol**	Pearson Correlation	1	0.159**	-0.038	0.588**
	Sig. (2-tailed)		<0.001	0.236	< 0.001
	Sum of Squares and Cross-products	180.996	33.025	-27.548	403.875
	Covariance	0.189	0.034	-0.029	0.421
	N	960	960	960	960
**Non-smokers vs. ever smokers**	Pearson Correlation	0.159**	1	-0.091**	0.121**
	Sig. (2-tailed)	<0.001		0.004	<0.001
	Sum of Squares and Cross-products	33.025	241.851	-76.289	95.694
	Covariance	.034	.250	-.079	.099
	N	960	968	968	968
**Total score diet**	Pearson Correlation	-.038	-.091**	1	-0.064*
	Sig. (2-tailed)	0.236	0.004		0.046
	Sum of Squares and Cross-products	-27.548	-76.289	2876.826	-175.983
	Covariance	-0.029	-0.079	2.975	-0.182
	N	960	968	968	968
**No. of drinks/week**	Pearson Correlation	0.588**	0.121**	-0.064*	1
	Sig. (2-tailed)	<0.001	<0.001	0.046	
	Sum of Squares and Cross-products	403.875	95.694	-175.983	2604.760
	Covariance	0.421	0.099	-0.182	2.694
	N	960	968	968	968

**. Correlation is significant at the 0.01 level (2-tailed).

*. Correlation is significant at the 0.05 level (2-tailed).

Supplementary Table S3. Distribution of the IHR score and health-related behaviours.

Smoking	All	Very low IHR	Low IHR	Medium IHR	Relatively high IHR	High IHR
Never smokers	496 (51.2%)	11 (57.9%)	9 (56.3%)	28 (40.6%)	80 (47.6%)	368 (52.9%)
Ex-smokers	81	2 (10.5%)	0 (0%)	12 (14.8%)	17 (21%)	50 (61.7%)
Current smokers	391 (40.4%)	6 (31.6%)	7 (43.8%)	29 (42%)	71 (42.3%)	278 (39.9%)
Alcohol consumption**						
Yes	242 (25.2%)	2 (10.5%)	2 (13.3%)	17 (24.6%)	62 (36.9%)	159 (23.1%)
No	718 (74.8%)	17 (89.55)	13 (86.7%)	52 (75.4%)	106 (63.1%)	530 (76.9%)
Diet components						
Red meet						
YES	361 (37.4%)	4 (21.1%)	4 (28.6%)	29 (42%)	63 (37.5%)	261 (37.6%)
NO	604 (62.65)	15 (78.9%)	10 (71.4%)	40 (58%)	105 (62.5%)	434 (62.4%)
Poultry						
YES	343 (35.5%)	5 (26.3%)	7 (50%)	24 (34.8%)	69 (41.1%)	238 (34.2%)
NO	623 (64.5%)	14 (73.7%)	7 (50%)	45 (65.2%)	99 (58.9%)	458 65.8%)
Processed meat						
YES	294 (30.4%)	3 (15.8%)	7 (46.7%)	23 (33.3%)	53 (31.5%)	208 (29.9%)
NO	672 (69.6%)	16 (84.2%)	8 (53.3%)	46 (66.7%)	115 (68.5%)	487 (70.1%)
Fish						
YES	338 (35%)	6 (31.6%)	4 (28.6%)	23 (33.3%)	60 (35.7%)	245 35.2%)
NO	628 (65%)	13 (68.4%)	10 (71.4%)	46 (66.7%)	108 (64.3%)	451 (64.8%)
Fruits						
YES	415 (43%)	7 (36.8%)	7 (46.7%)	30 (43.5%)	66 (39.3%)	305 (43.9%)
NO	550 (57%)	12 (63.2%)	8 (53.3%)	39 (56.5%)	102 (60.7%)	389 (56.1%)
Vegetable (cooked)	0.514					
YES	195 (20.2%)	3 (15.8%)	4 (25%)	9 (13%)	38 (22.6%)	141 (20.3%)
NO	772 (79.8%)	16 (84.2%)	12 (75%)	60 (87%)	130 (77.4%)	554 (79.7%)
Vegetable (raw)						
YES	410 (42.5%)	6 (31.6%)	5 (33.3%)	27 (39.1%)	72 (42.9%)	300 (43.3%)
NO	554 (57.5%)	13 (68.4%)	10 (66.7%)	42 (60.9%)	96 (57.1%)	393 (56.7%)
Dairy	0.678					
YES	436 (45.2%)	8 (42.1%)	8 (57.1%)	27 (39.1%)	73 (43.5%)	320 (46%)
NO	529 (54.8%)	11 (57.9%)	6 (42.9%)	42 (60.9%)	95 (56.5%)	375 (54%)
Cookies	0.584					
YES	81 (8.4%)	0 (0%)	2 (13.3%)	7 (10.1%)	16 (9.5%)	56 (8.1%)
NO	882 (91.6%)	19 (100%)	13 (86.7%)	62 (89.9%)	152 (90.5%)	636 (91.9%)
Soft drinks						
YES	411 (42.6%)	7 (36.8%)	7 (43.8%)	24 (34.8%)	68 (40.7%)	305 (43.9%)
NO	554 (57.4%)	12 (63.2%)	9 (56.3%)	45 (65.2%)	99 (59.3%)	389 (56.1%)
Screening test participation						
YES	771 (79.6%)	13 (68.4%)	12 (75%)	53 (76.8%)	142 (84.5%)	551 (79.2%)
NO	197 (20.4%)	6 (31.6%)	4 (25%)	16 (23.2%)	26 (15.5%)	145 (20.8%)

** $p<0.05$; *** $p<0.001$

Supplementary Table S4. Average No. of drinks/week according to IHR score.

IHR	Mean	N	Std. Deviation
1	.16	19	.501
2	.31	16	1.014
3	.46	69	1.023
4	1.18	168	3.039
5	.43	696	1.132
Total	.56	968	1.641

Supplementary Table S5. Pairwise comparisons of No. of drinks in different categories of IHR.

Sample 1-Sample 2	Test Statistic	Std. Error	Std. Test Statistic	Sig.	Adj. Sig.[a]
1-2	-13.179	71.454	-0.184	0.854	1.000
1-5	-58.300	48.967	-1.191	0.234	1.000
1-3	-63.950	54.559	-1.172	0.241	1.000
1-4	-129.737	50.970	-2.545	0.011	0.109
2-5	-45.120	53.248	-0.847	0.397	1.000
2-3	-50.770	58.432	-0.869	0.385	1.000
2-4	-116.558	55.096	-2.116	0.034	0.344
5-3	5.650	26.578	0.213	0.832	1.000
5-4	71.438	18.102	3.946	<0.001	0.001
3-4	-65.788	30.111	-2.185	0.029	0.289

Each row tests the null hypothesis that the Sample 1 and Sample 2 distributions are the same.

Asymptotic significances (2-sided tests) are displayed. The significance level is .05.

a. Significance values have been adjusted by the Bonferroni correction for multiple tests.

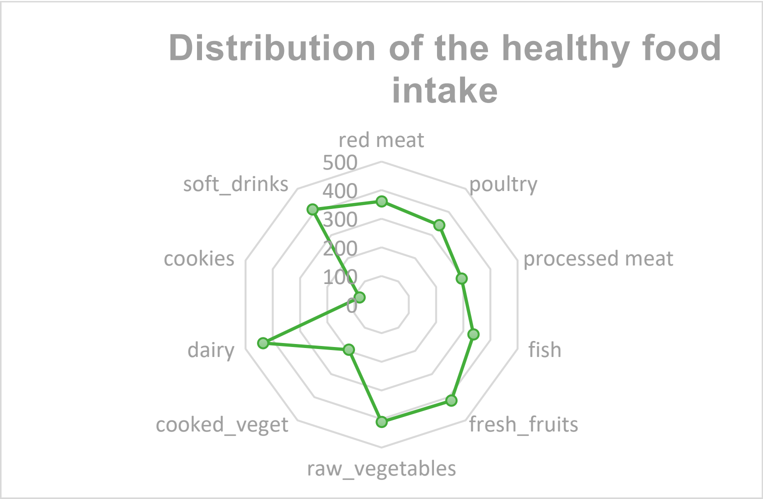

Figure S1. Number of respondents who complied with the recommended consumption frequency by food category.
